# Impact of Underweight after Treatment on Prognosis of Advanced-Stage Ovarian Cancer

**DOI:** 10.1155/2014/349546

**Published:** 2014-06-24

**Authors:** Se Ik Kim, Hee Seung Kim, Tae Hun Kim, Dong Hoon Suh, Kidong Kim, Jae Hong No, Hyun Hoon Chung, Yong Beom Kim, Yong Sang Song

**Affiliations:** ^1^Department of Obstetrics and Gynecology, Seoul National University College of Medicine, Seoul 110-744, Republic of Korea; ^2^Department of Obstetrics and Gynecology, Korean Cancer Center Hospital, Korea Institute of Radiological and Medical Sciences, Seoul 139-706, Republic of Korea; ^3^Department of Obstetrics and Gynecology, Seoul National University Bundang Hospital, Seongnam 463-707, Republic of Korea; ^4^Cancer Research Institute, Seoul National University College of Medicine, Seoul 110-799, Republic of Korea; ^5^World Class University, Seoul National University, Seoul 151-921, Republic of Korea

## Abstract

This study aimed to investigate the impact of underweight status on the prognosis of advanced-stage ovarian cancer. A total of 360 patients with stage III-IV epithelial ovarian cancer were enrolled and divided into three groups by body mass indexes (BMIs): underweight (BMI < 18.5 kg/m^2^); normal weight to overweight (18.5 kg/m^2^ BMI < 27.5 kg/m^2^); obesity (BMI ≥ 27.5 kg/m^2^). Progression-free survival (PFS), overall survival (OS), CA-125, and neutrophil to lymphocyte ratio (NLR) as a marker reflecting host inflammation and immunity were compared among the three groups according to the three treatment times: at diagnosis; after surgery; and after treatment. Only underweight status after treatment was associated with poor OS in comparison with normal weight to overweight or obesity (mean value, 44.9 versus 78.8 or 67.4 months; *P* = 0.05); it was also an unfavorable factor for OS (adjusted HR, 2.29; 95% CI, 1.08–4.85). Furthermore, NLR was higher in patients with underweight than in those with obesity after treatment (median value, 2.15 versus 1.47; *P* = 0.03), in spite of no difference in CA-125 among the three groups at the three treatment times. In conclusion, underweight status after treatment may be a poor prognostic factor in patients with advanced-stage ovarian cancer, which accompanies increased host inflammation and decreased immunity.

## 1. Introduction

Excessive bodyweight is an established risk factor for several types of cancer. In particular, epidemiologic data show that obesity defined as body mass index (BMI) ≥ 30 kg/m^2^ increases cancer risk and cancer-specific mortality [1, 2]. Although the precise mechanism is not clear, some obesity-related changes are expected to contribute to an increased risk of cancer. Insulin resistance and hyperinsulinemia are commonly observed in obesity. In this condition, secretion of insulin-like growth factor-1 (IGF-1) and various cytokines, such as adipokines, are stimulated. These factors promote cell proliferation, cell survival, and angiogenesis [3, 4]. Moreover, reactive oxygen radicals, increased by obesity, lead to systemic inflammation contributing to cancer development [5]. Recent epidemiologic studies supported these mechanisms, suggesting that obesity may affect poor prognosis in some cancers [6, 7].

However, the impact of underweight status on prognosis has not been adequately addressed. Although underweight status has been reported to be a high-risk factor for recurrence and death in patient with breast cancer [8], its role has not been evaluated in ovarian cancer. Furthermore, even in a recent meta-analysis, which showed slightly worse survival in obesity patients with ovarian cancer, the impact of BMI including underweight status, as well as obesity, was unclear because of a large amount of interstudy variation [9, 10].

Therefore, we investigated the impact of underweight status on prognosis in patients with advanced-stage ovarian cancer, depending on the time of measurement of BMI in relation to the treatment. Thus, we evaluated the relationship between underweight status and cancer progression, with related changes of systemic inflammation and immunity.

## 2. Materials and Methods

### 2.1. Study Population

Clinicopathologic data for the current study were retrieved from a database of 360 patients registered in two tertiary medical centers (Seoul National University Hospital and Seoul National University Bundang Hospital) between 2000 and 2011. The current study was approved by the Institutional Review Board of Seoul National University Hospital. The patients' medical records were reviewed retrospectively. Informed consent was not required since the current study was conducted by a retrospective review of medical records.

### 2.2. Inclusion or Exclusion Criteria

We included patients with the following inclusion criteria: those with epithelial ovarian cancer; those with advanced-stage disease, in particular, the International Federation of Gynecology and Obstetrics (FIGO) stage III-IV disease; those who underwent staging operation and taxane- and platinum-based chemotherapy; those with BMIs measured at three treatment points including “at diagnosis,” “after surgery,” and “after treatment.” We excluded patients with nonepithelial ovarian cancer, synchronous or metachronous cancer, and insufficient data for investigating the impact of BMI on survival.

### 2.3. Data Collection

BMIs at diagnosis, after surgery, and after treatment were defined as those measured at diagnosis, before the first administration of adjuvant chemotherapy, and after the last administration of adjuvant chemotherapy. Furthermore, all patients were classified into four groups based on the following BMI criteria suggested by the World Health Organization for the Asian population: underweight (BMI < 18.5 kg/m^2^); normal (18.5 kg/m^2^ ≤ BMI < 23.0 kg/m^2^); overweight (23.0 kg/m^2^ ≤ BMI < 27.5 kg/m^2^); and obesity (BMI ≥ 27.5 kg/m^2^) [11].

To evaluate the potential of cancer progression and related changes of systemic inflammation and immunity, serum CA-125 level and neutrophil to lymphocyte ratio (NLR) were investigated. NLR is known as a prognostic factor for recurrence and death in patients with ovarian cancer [12, 13]. Since increased inflammation and decreased immunity by cancer contribute to secondary hematological changes, including relative neutrophilia and lymphocytopenia, NLR tends to increase in several types of malignancy [14, 15]. Thus, we measured CA-125 as a tumor marker and NLR as a marker of systemic inflammation and immunity, using a radioimmunoassay kit (Fujirebio Diagnostics, Malvern, PA, USA) and SYSMEX XE-2100 (TOA Medical Electronics, Kobe, Japan) at diagnosis, after surgery, and after treatment, respectively.

Clinicopathologic characteristics including age, grade, FIGO stage, histology, neoadjuvant chemotherapy, cycles of adjuvant chemotherapy, optimal debulking surgery, progression-free survival (PFS), and overall survival (OS) were collected. Patients treated with neoadjuvant chemotherapy received three cycles of taxane- and platinum-based chemotherapy before surgery, and optimal debulking surgery was considered when the size of residual tumor was less than 1 cm in the longest diameter. PFS was defined as the time that elapsed from the date after completion of the primary treatment to the date of clinically proven recurrence. OS was defined as the time that elapsed from the date of diagnosis to the date of cancer-related death or end of the study.

### 2.4. Statistical Methods

Kruskal-Wallis, Mann-Whitney *U*, and Chi-square tests were used to determine differences in clinicopathologic characteristics among underweight, normal to overweight, and obesity patients. Furthermore, univariate and multivariate analyses for investigating factors affecting survival were performed using the Kaplan-Meier method with log-rank test and Cox's proportional hazard regression model with hazard ratio (HR) and 95% confidence interval (CI). We conducted these statistical analyses using SPSS software (version 19.0; SPSS Inc., Chicago, IL, USA). A *P* < 0.05 was considered statistically significant.

## 3. Results

### 3.1. Patients' Characteristics

Clinicopathologic characteristics of all patients are depicted in Supplementary Table 1 (see Supplementary Material available online at http://dx.doi.org/10.1155/2014/349546). The mean age was 53.9 years (range, 18–80 years) and 5 (1.4%), 23 (6.4%), 256 (71.1%), and 76 (21.1%) patients had stage IIIA, IIIB, IIIC, and IV diseases, respectively. Furthermore, serous carcinoma was identified in 276 (76.7%) patients while endometrioid, clear cell, mucinous, undifferentiated, and mixed carcinomas were observed in 29 (8.1%), 20 (5.6%), 13 (3.6%), 7 (1.9%), and 15 (4.2%), respectively. Three cycles of neoadjuvant chemotherapy using taxane and platinum were administered in 57 patients (15.8%), and the mean value of cycles of adjuvant chemotherapy using the same regimen was 6 (range, 3–12).

**Figure 1:**
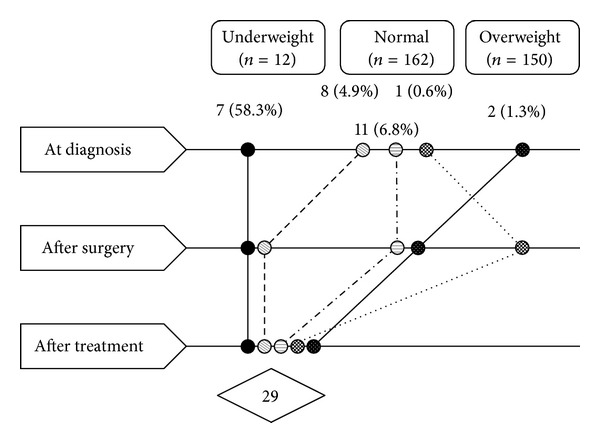
Underweight patients with advanced-stage ovarian cancer according to the treatment time.

Among 360 patients, the following conditions were identified: underweight, normal, overweight, and obesity in 12 (3.3%), 162 (45.0%), 150 (41.7%), and 36 (10.0%) patients, respectively,* at diagnosis*; 32 (8.9%), 183 (50.8%), 118 (32.8%), and 27 (7.5%) patients, respectively,* after surgery*; 29 (8.1%), 146 (40.6%), 157 (43.6%), and 28 (7.8%) patients, respectively,* after treatment*. In particular, 7 patients (58.3%) who showed underweight status at diagnosis were underweight even at the after treatment time point (Figure 1).

After treatment, patients with hypertension were observed in 2 out of 29 underweight (6.9%), 20 out of 146 normal (13.7%), 19 out of 157 overweight (12.1%), and 13 out of 28 obesity (46.4%). The prevalence of hypertension significantly increased as the patient's BMI after treatment increased toward obesity (*P* = 0.003). After treatment, patients with diabetes were observed in 9 out of 146 normal (6.2%), 10 out of 157 overweight (6.4%), and 2 out of 28 obesity (7.1%). The prevalence of diabetes had the same trends, but without statistical significance (*P* = 0.372).

### 3.2. Underweight Status Effects on Prognosis or Systemic Inflammation and Immunity

**Figure 2:**
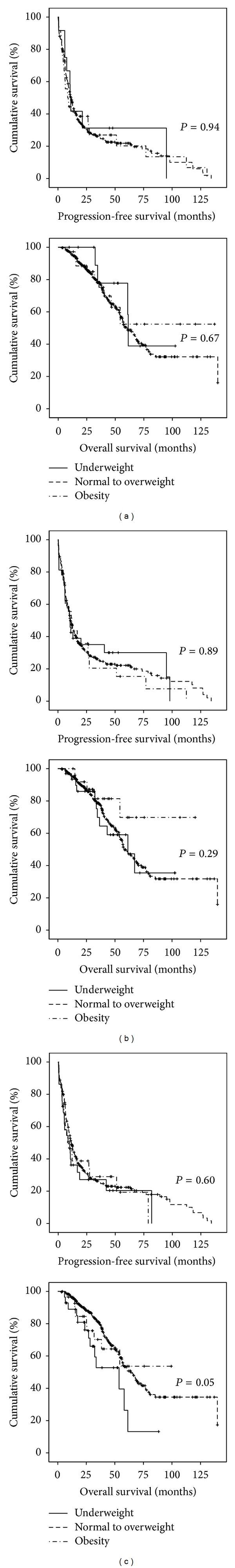
Kaplan-Meier analyses with the log-rank test for comparing progression-free survival and overall survival among patients with underweight, normal to overweight, and obesity with advanced-stage ovarian cancer: (a) at diagnosis; (b) after surgery; (c) after treatment.

We compared PFS and OS among underweight, normal to overweight, and obesity patients, according to the treatment time. As a result, only patients with underweight status* after treatment *showed poor OS in comparison with those with normal to overweight or obesity (mean value, 44.9 versus 78.8 or 67.4 months; *P* = 0.05; Figure 2). When we adjusted the result with clinicopathologic characteristics, underweight status after treatment was an unfavorable factor for OS (adjusted HR, 2.29; 95% CI, 1.08–4.85; Table 1).

Next, we compared CA-125 and NLR among underweight, normal to overweight, and obesity patients according to the treatment time (Table 2). As a result, CA-125* at diagnosis* was higher in patients with normal to overweight status or obesity than in those with underweight status (median value, 865 or 912.5 versus 185.5 U/mL; *P* = 0.04). Since underweight patients* at diagnosis* achieved more frequent optimal debulking surgery than those with normal weight to overweight or obesity, we did subgroup analyses based on whether optimal debulking surgery was performed. As a result, there were no differences in CA-125 and NLR among underweight, normal to overweight, and obesity patients who underwent optimal debulking surgery (median value of CA-125, 161.5 versus 555 versus 490 U/mL; *P* = 0.37: median value of NLR, 2.93 versus 2.51 versus 2.54; *P* = 0.86) and suboptimal debulking surgery (median value of CA-125, 956 versus 1,043 versus 930 U/mL; *P* = 0.68: median value of NLR, 3.29 versus 3.49 versus 3.43; *P* = 0.55).

Furthermore, the rate of optimal debulking surgery was also different between underweight and obesity patient groups* after surgery*, in spite of no differences of CA-125 and NLR. Thus, we also did subgroup analyses according to whether optimal debulking surgery was performed and observed that CA-125 and NLR were not different among underweight, normal to overweight, and obesity patients who underwent optimal debulking surgery (median value of CA-125, 71 versus 76.5 versus 65 U/mL; *P* = 0.39: median value of NLR, 2.58 versus 2.37 versus 2.68; *P* = 0.43) and suboptimal debulking surgery (median value of CA-125, 216.8 versus 202 versus 105 U/mL; *P* = 0.52: median value of NLR, 2.53 versus 3.05 versus 3.71; *P* = 0.15).

On the other hand, underweight patients* after treatment* showed higher NLR than those with obesity, in spite of no differences of confounding factors between two groups (median value, 2.15 versus 1.47; *P* = 0.03).

### 3.3. Degree of Weight Loss Effects on Prognosis in Underweight Patients after Treatment

Next, we compared clinicopathologic characteristics and prognosis according to the degree of weight loss, only in patients with underweight status* after treatment*. All 29 patients with underweight status* after treatment* were divided into two subgroups according to the following criteria: weight loss ≥10% versus <10% from the body weight at diagnosis. Clinicopathologic characteristics based on the degree of weight loss are summarized in Supplementary Table 2. Although there were no differences in age, FIGO stage, histology, grade, neoadjuvant chemotherapy, cycles of adjuvant chemotherapy, CA-125, and NLR between two subgroups, the success rate of optimal debulking surgery was higher in underweight patients with weight loss <10% than in those with weight loss ≥10% (83.3% versus 36.4%; *P* = 0.02).

**Figure 3:**
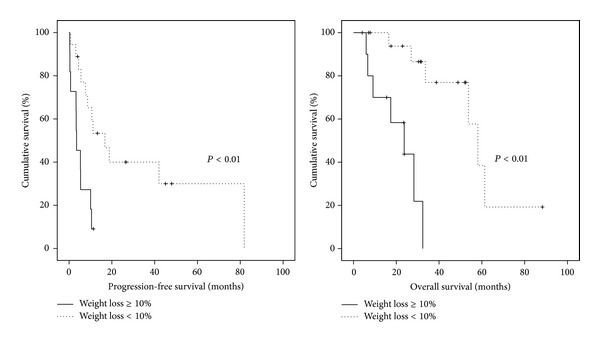
Kaplan-Meier analyses with the log-rank test for comparing progression-free survival and overall survival between weight loss ≥10% and <10% in underweight patients after treatment.

Furthermore, underweight patients with weight loss ≥10% showed poor PFS and OS in comparison with those with weight loss <10% (PFS, median value, 3.5 versus 16.8 months; OS, median value, 23.7 versus 58.1 months; Figure 3). Weight loss ≥10% was also a poor prognostic factor for PFS and OS when adjusted with other clinicopathologic factors (adjusted HRs, 6.90 and 15.27; 95% CIs, 1.51–31.54 and 1.42–164.5; Table 3).

## 4. Discussion

In terms of the association between BMI and cancer risk and prognosis, most of the studies have focused mainly on the impact of obesity, because deleterious mechanisms related to obesity are expected to be unfavorable to cancer patients [16]. The metabolic syndrome, a cluster of risk factors for cardiovascular disease and type 2 diabetes, is considered to play a central role in this relationship [17–19].

However, excessive weight loss can also be associated with poor prognosis, because it has similar features to cancer cachexia, a complex metabolic condition characterized by loss of skeletal muscle and body weight, developed in progressive disease [20, 21].

Epidemiologically, the relationship between the risk of mortality and BMI is known to be U-shaped with the increased risk related to either cachexia showing very low BMI or obesity demonstrating very high BMI, whereas emerging data indicate that obesity is associated paradoxically with better prognosis in cancer patients [22]. In the current study, we also found that underweight status after treatment was an unfavorable factor for OS in patients with advanced-stage ovarian cancer (adjusted HR, 2.29; 95% CI, 1.08–4.85), whereas obesity was not associated with prognosis, regardless of the treatment time. The lack of an association between obesity and prognosis can be explained by the following reasons. The cut-off value defining obesity is relatively lower in the Asian population than in the Western population (27.5 kg/m^2^ versus 30 kg/m^2^), and it may result in different effects of obesity based on race. Moreover, obesity can help patients endure the increased resting energy expenditure (REE) which occurs in cancer [20]. This endurance can help patients with advanced-stage ovarian cancer, with a 5-year survival rate of approximately 30% [23], to maintain their general condition thereby improving survival. These hypotheses were supported by recent epidemiologic data showing no association between obesity and poor prognosis in Asian patients with ovarian cancer [24].

However, underweight status can act as a poor prognostic factor in these patients. Theoretically, most patients with advanced-stage ovarian cancer should recover from their underweight status after treatment because the Warburg effect, that is, increased glucose uptake by tumors for glycolysis to generate ATP, is expected to reduce with the decrease of tumor burden after treatment [25]. Inversely, failure to regain weight after treatment indicates that the cancer has potentially progressed, and it is easily identified in patients with cancer cachexia. In cancer cachexia, systemic inflammation is induced and persists due to increased tumor necrosis factor-alpha (TNF-*α*), interleukin-6 (IL-6), IL-1, and interferon-gamma (IFN-*γ*). This results in decrease of protein anabolism and caloric intake, while promoting increase of protein catabolism, insulin resistance, lipolysis, and REE. Eventually, loss of muscle mass and strength, loss of whole body fat, ineffective host's antitumor response, and impaired immunity occur and lead patients to physical disability, diminished quality of life, and reduced survival [20, 26–29].

To prove this hypothesis clinically, we investigated CA-125 as a tumor marker and NLR as a marker reflecting inflammation and immunity among underweight, normal weight to overweight, and obesity patients according to the treatment time. After treatment, although there were no differences of CA-125 among the three groups, underweight patients showed the highest NLR compared with normal weight to overweight and obesity patients, suggesting increased systemic inflammation (neutrophilia) and decreased immunity (lymphocytopenia) in these patients. This means that underweight status after treatment is a condition which increases the likelihood of cancer progression, and it can be considered as an early marker for poor prognosis in patients with advanced-stage ovarian cancer.

Chronic systemic inflammation is also known to be related to metabolic syndrome, which is in state of central obesity or excessive adiposity [30]. However, in the current study, underweight patients after treatment showed relatively higher increase in systemic inflammation compared to obesity patients. This can be explained as follows. First, inflammatory state of underweight patients may reflect ethnic variation. The Asian subjects are known to develop metabolic syndrome at a relatively low level of BMI compared to the Western populations [31], which can be explained by ethnic variations in body fat distribution [32]. Second, BMI alone does not exactly predict fat distribution and adiposity in individuals. Third, although the prevalence of metabolic syndrome in the enrolled patients was not known due to the lack of data, including waist circumference and serum levels of triglyceride or high density lipoprotein, patients with hypertension and/or diabetes were more common in obesity patients and all of them were under adequate and specific medications. The antihypertensive and/or antidiabetic drugs could counterbalance the underlying proinflammatory state which was generated from metabolic syndrome [33]. Lastly, underweight patients after treatment in advanced-stage ovarian cancer include patients with cancer cachexia, which is well known to have chronic systemic inflammation that can result in poor prognosis [26–29].

In terms of cancer cachexia, it was hard to assess exactly how many underweight patients after treatment were in cachectic state according to the retrospective analysis of medical records. To consider cancer patients to be in cachectic state, at least all three key features of cachexia should be presented as follows: weight loss >10%; systemic inflammation (C-reactive protein (CRP) > 10 mg/L); and reduced food intake (<1,500 kcal/day) [20]. However, serum CRP levels and food intake and/or nutritional status of patients have not been routinely observed in our institute. Only the change in each patient's weight from the diagnosis was able to be retrieved.

Thus, we divided all underweight patients after treatment into two subgroups on the basis of weight loss by 10% from the bodyweight at diagnosis, considering the features of cancer cachexia. As a result, the risk of suboptimal surgery increased in underweight patients with weight loss ≥10% (63.6% versus 16.7%; *P* = 0.02), and weight loss ≥10% was an independent poor prognostic factor for PFS and OS (adjusted HRs, 6.90 and 15.27; 95% CIs, 1.51–31.54 and 1.42–164.5). These data indicated that severe weight loss (≥10%) after treatment was associated with more unresectable tumors and an increased risk of cancer progression. However, there was no difference in NLR between the two subgroups in spite of the tendency that it was higher in underweight patients with weight loss ≥10% than in those with weight loss <10% (median value, 2.15 versus 2.04). The small number of underweight patients enrolled in the study likely led to no statistical difference; a large-scale cohort is needed in future studies.

The current study is the first report demonstrating the impact of underweight status after treatment on prognosis of gynecologic cancer. However, there were some limitations. Firstly, we could not evaluate the impact of underweight status on prognosis of patients with early-stage ovarian cancer, because they showed good prognosis. Secondly, we measured only NLR as an indicator of host inflammation and immunity because other proinflammatory markers or cytokines were not included in the clinical setting. Thirdly, all patients in the current study were ethnically homogenous Asians, so the results may not be applicable to other ethnic groups.

In conclusion, we found that underweight status after treatment may be a poor prognostic factor in patients with advanced-stage ovarian cancer, and it is accompanied by increased tumor-induced inflammation and decreased immunity. Underweight status can act as an early marker to predict poor prognosis. In particular, paying attention to weight change is required during the treatment period, because more than half of underweight patients at diagnosis failed to gain weight and a weight loss ≥10% after treatment was associated with an increased risk of disease recurrence and mortality.

## Supplementary Material

Supplementary Table 1: Clinicopathologic characteristics of 360 patients with advanced-stage ovarian cancer.
Supplementary Table 2: Clinicopathologic characteristics of 29 underweight patients after treatment according to the degree of weight loss.

## Figures and Tables

**Table 1:** Clinicopathologic factors affecting progression-free and overall survivals in all 360 patients with advanced-stage ovarian cancer.

Characteristics	Univariate			Multivariate		
	HR	95% CI	*P* value	Adjusted HR	95% CI	*P* value
Progression-free survival						
≥53 years	1.04	0.82–1.32	0.74	—	—	—
Stage IV disease	1.27	0.95–1.69	0.11	—	—	—
Grade 3 disease	1.11	0.80–1.53	0.53	—	—	—
Nonserous histology	1.26	0.95–1.67	0.11	—	—	—
No neoadjuvant chemotherapy	1.62	1.19–2.20	<0.01	1.84	1.18–2.87	<0.01
≤6 cycles of adjuvant chemotherapy	1.03	0.81–1.32	0.81	—	—	—
Suboptimal debulking surgery	1.54	1.21–1.96	<0.01	1.71	1.22–2.39	<0.01
Underweight after treatment	1.25	0.80–1.93	0.33	—	—	—
Overall survival						
≥53 years	1.12	0.79–1.58	0.52	—	—	—
Stage IV disease	1.21	0.80–1.84	0.36	—	—	—
Grade 3 disease	1.21	0.75–1.93	0.44	—	—	—
Nonserous histology	1.58	1.07–2.33	0.02	—	—	—
No neoadjuvant chemotherapy	1.65	1.08–2.54	0.02	1.88	1.28–2.77	<0.01
≤6 cycles of adjuvant chemotherapy	1.16	0.82–1.64	0.42	—	—	—
Suboptimal debulking surgery	1.49	1.05–2.11	0.03	1.67	1.23–2.28	<0.01
Underweight after treatment	2.01	1.13–3.58	0.02	2.29	1.08–4.85	0.03

**Table 2:** Comparison of CA-125 and neutrophil to lymphocyte ratio (NLR) among underweight, normal to overweight, and obesity patients according to the treatment time.

BMI	CA-125 (median, U/mL)	NLR (median)	Confounding factors			
			Stage IV disease	Grade 3 disease	Nonserous histology	Suboptimal debulking
At diagnosis						
Underweight	185.5	8^∗,†^	1 (8.3)^∗,†^	3 (25.0)^∗,†^	4 (33.3)^∗,†^	2 (16.7)∗
Normal to overweight	865∗	9^∗,‡^	69 (22.3)^∗,‡^	156 (50.3)^∗,‡^	69 (22.3)^∗,‡^	149 (48.1)^∗,†^
Obesity	912.5∗	6.75^†,‡^	5 (13.9)^†,‡^	21 (58.3)^†,‡^	5 (13.9)^†,‡^	21 (58.3)^†^
*P* value	0.04	0.47	0.28	0.14	0.33	0.04
After surgery						
Underweight	95.6^∗,†^	2.58^∗,†^	6 (18.8)^∗,†^	15 (46.9)^∗,†^	3 (9.4)^∗,†^	10 (32.2)^∗,†^
Normal to overweight	190.0^∗,‡^	2.67^∗,‡^	65 (22.3)^∗,‡^	146 (50)^∗,‡^	67 (22.9)^∗,‡^	143 (49)^∗,‡^
Obesity	87.5^†,‡^	3.14^†,‡^	5 (18.5)^†,‡^	16 (59.3)^†,‡^	5 (17.9)^†,‡^	16 (59.3)^†,‡^
*P* value	0.78	0.23	0.83	0.60	0.29	0.08
After treatment						
Underweight	8^∗,†^	2.15∗	5 (17.2)^∗,†^	13 (44.8)^∗,†^	6 (20.7)^∗,†^	10 (34.5)^∗,†^
Normal to overweight	9^∗,‡^	1.56^∗,†^	62 (20.7)^∗,‡^	148 (50.7)^∗,‡^	67 (22.4)^∗,‡^	146 (48.8)^∗,‡^
Obesity	6.8^†,‡^	1.47^†^	8 (28.6)^†,‡^	18 (66.7)^†,‡^	4 (14.3)^†,‡^	13 (46.4)^†,‡^
*P* value	0.21	0.09	0.54	0.27	0.60	0.33

BMI: body mass index; ^∗,†,‡^no significant difference between two groups with the same symbol.

**Table 3:** Clinicopathologic factors affecting progression-free and overall survivals in 29 patients who showed underweight after treatment.

Characteristics	Univariate			Multivariate		
	HR	95% CI	*P* value	Adjusted HR	95% CI	*P* value
Progression-free survival						
≥53 years	1.91	0.79–4.63	0.15	—	—	—
Stage IV disease	1.88	0.68–5.18	0.23	4.89	1.14–20.94	0.03
Grade 3 disease	0.94	0.33–2.70	0.91	—	—	—
Nonserous histology	2.99	1.13–7.90	0.03	—	—	—
No neoadjuvant chemotherapy	1.54	0.51–4.64	0.45	—	—	—
≤6 cycles of adjuvant chemotherapy	1.10	0.40–3.01	0.86	—	—	—
Suboptimal debulking surgery	3.89	1.55–9.74	<0.01	10.04	1.48–68.13	0.02
Weight loss ≥10%	4.07	1.55–10.64	<0.01	6.90	1.51–31.54	0.01
Overall survival						
≥53 years	2.07	0.62–6.92	0.24	—	—	—
Stage IV disease	1.92	0.40–9.25	0.42	11.9	1.00–141.1	0.05
Grade 3 disease	0.77	0.17–3.47	0.73	—	—	—
Nonserous histology	2.58	0.73–9.15	0.14	—	—	—
No neoadjuvant chemotherapy	1.27	0.27–6.02	0.77	—	—	—
≤6 cycles of adjuvant chemotherapy	3.84	0.78–18.91	0.10	—	—	—
Suboptimal debulking surgery	2.64	0.88–7.91	0.08	—	—	—
Weight loss ≥10%	12.81	2.54–64.65	<0.01	15.27	1.42–164.5	0.02

## References

[1] Renehan AG, Tyson M, Egger M, Heller RF, Zwahlen M (2008). Body-mass index and incidence of cancer: a systematic review and meta-analysis of prospective observational studies. *The Lancet*.

[2] Gilbert CA, Slingerland JM (2013). Cytokines, obesity, and cancer: new insights on mechanisms linking obesity to cancer risk and progression. *Annual Review of Medicine*.

[3] Cowey S, Hardy RW (2006). The metabolic syndrome: a high-risk state for cancer?. *The American Journal of Pathology*.

[4] Calle EE, Kaaks R (2004). Overweight, obesity and cancer: epidemiological evidence and proposed mechanisms. *Nature Reviews Cancer*.

[5] Gago-Dominguez M, Castelao JE, Yuan J, Ross RK, Yu MC (2002). Lipid peroxidation: a novel and unifying concept of the etiology of renal cell carcinoma (United States). *Cancer Causes & Control*.

[6] Sparano JA, Wang M, Zhao F (2012). Obesity at diagnosis is associated with inferior outcomes in hormone receptor-positive operable breast cancer. *Cancer*.

[7] Ho T, Gerber L, Aronson WJ (2012). Obesity, prostate-specific antigen nadir, and biochemical recurrence after radical prostatectomy: biology or technique? Results from the SEARCH database. *European Urology*.

[8] Moon HG, Han W, Noh DY (2009). Underweight and breast cancer recurrence and death: a report from the Korean Breast Cancer Society. *Journal of Clinical Oncology*.

[9] Protani MM, Nagle CM, Webb PM (2012). Obesity and ovarian cancer survival: a systematic review and meta-analysis. *Cancer Prevention Research*.

[10] Suh DH, Kim J, Kim K, Kim HJ, Lee K (2012). Major clinical research advances in gynecologic cancer in 2012. *Journal of Gynecologic Oncology*.

[11] Claise B

[12] Cho H, Hur HW, Kim SW (2009). Pre-treatment neutrophil to lymphocyte ratio is elevated in epithelial ovarian cancer and predicts survival after treatment. *Cancer Immunology, Immunotherapy*.

[13] Thavaramara T, Phaloprakarn C, Tangjitgamol S, Manusirivithaya S (2011). Role of neutrophil to lymphocyte ratio as a prognostic indicator for epithelial ovarian cancer. *Journal of the Medical Association of Thailand*.

[14] Kim HS, Han KH, Chung HH (2010). Neutrophil to lymphocyte ratio for preoperative diagnosis of uterine sarcomas: a case-matched comparison. *European Journal of Surgical Oncology*.

[15] Walsh SR, Cook EJ, Goulder F, Justin TA, Keeling NJ (2005). Neutrophil-lymphocyte ratio as a prognostic factor in colorectal cancer. *Journal of Surgical Oncology*.

[16] Louie SM, Roberts LS, Nomura DK (2013). Mechanisms linking obesity and cancer. *Biochimica et Biophysica Acta*.

[17] Alberti KG, Eckel RH, Grundy SM (2009). Harmonizing the metabolic syndrome: a joint interim statement of the international diabetes federation task force on epidemiology and prevention; National heart, lung, and blood institute; American heart association; World heart federation; International atherosclerosis society; and international association for the study of obesity. *Circulation*.

[18] Zhou JR, Blackburn GL, Walker WA (2007). Symposium introduction: metabolic syndrome and the onset of cancer. *The American Journal of Clinical Nutrition*.

[19] Nicolucci A (2010). Epidemiological aspects of neoplasms in diabetes. *Acta Diabetologica*.

[20] Tan BHL, Fearon KCH (2008). Cachexia: prevalence and impact in medicine. *Current Opinion in Clinical Nutrition and Metabolic Care*.

[21] Morley JE, Thomas DR, Wilson MM (2006). Cachexia: pathophysiology and clinical relevance. *The American Journal of Clinical Nutrition*.

[22] Kalantar-Zadeh K, Horwich TB, Oreopoulos A (2007). Risk factor paradox in wasting diseases. *Current Opinion in Clinical Nutrition and Metabolic Care*.

[23] Heintz AP, Odicino F, Maisonneuve P Carcinoma of the ovary. FIGO 26th Annual Report on the Results of Treatment in Gynecological Cancer. *International Journal of Gynaecology and Obstetrics*.

[24] Suh DH, Kim HS, Chung HH (2012). Body mass index and survival in patients with epithelial ovarian cancer. *Journal of Obstetrics and Gynaecology Research*.

[25] Tisdale MJ (2009). Mechanisms of cancer cachexia. *Physiological Reviews*.

[26] Evans WJ, Morley JE, Argilés J (2008). Cachexia: a new definition. *Clinical Nutrition*.

[27] Evans WJ (2010). Skeletal muscle loss: cachexia, sarcopenia, and inactivity. *The American Journal of Clinical Nutrition*.

[28] Argilés JM, Busquets S, Felipe A, López-Soriano FJ (2005). Molecular mechanisms involved in muscle wasting in cancer and ageing: cachexia versus sarcopenia. *The International Journal of Biochemistry & Cell Biology*.

[29] Gullett N, Rossi P, Kucuk O, Johnstone PAS (2009). Cancer-induced cachexia: a guide for the oncologist. *Journal of the Society for Integrative Oncology*.

[30] Harvey AE, Lashinger LM, Hursting SD (2011). The growing challenge of obesity and cancer: an inflammatory issue. *Annals of the New York Academy of Sciences*.

[31] Seidell JC (2000). Obesity, insulin resistance and diabetes—a worldwide epidemic. *British Journal of Nutrition*.

[32] Raji A, Seely EW, Arky RA, Simonson DC (2001). Body fat distribution and insulin resistance in healthy Asian Indians and Caucasians. *The Journal of Clinical Endocrinology and Metabolism*.

[33] Karelis AD, St-Pierre DH, Conus F, Rabasa-Lhoret R, Poehlman ET (2004). Metabolic and body composition factors in subgroups of obesity: what do we know?. *The Journal of Clinical Endocrinology and Metabolism*.

