# Supplementary material for: Impact of Underweight after Treatment on Prognosis of Advanced-Stage Ovarian Cancer
Source: J Immunol Res. 2014 Jun 24;2014:349546. doi: 10.1155/2014/349546 (PMC4094724; doi:10.1155/2014/349546)
Supplement: Supplementary file 1 — Supplementary Table 1: Clinicopathologic characteristics of 360 patients with advanced-stage ovarian cancer. Supplementary Table 2: Clinicopathologic characteristics of 29 underweight patients after treatment according to the degree of weight loss. [file 349546.f1.pdf]

Supplementary Table 1. Clinico-pathologic characteristics of 360 patients with advanced-stage ovarian cancer

Characteristics	No. of Patients (%)
Age (y)	
<53	170 (47.2)
≥53	190 (52.8)
FIGO stage	
IIII	284 (78.9)
IV	76 (21.1)
Histology	
Serous	276 (76.7)
Non-serous	84 (23.3)
Grade	
1	10 (2.8)
2	59 (16.4)
3	181 (50.3)
Unknown	110 (30.6)
Residual tumor (cm)	
≥1	173 (48.1)
<1	187 (51.9)
Cycles of adjuvant chemotherapy	
≤6	224 (62.2)
>6	136 (37.8)

FIGO, International Federation of Gynecology and Obstetrics.

Supplementary Table 2. Clinico-pathologic characteristics of 29 underweight patients after treatment according to the degree of weight loss

Characteristics	Weight loss ≥10% (n=11, %)	Weight loss <10% (n=18, %)	P value
Age (y)			0.70
<53	7 (63.6)	9 (50)	
≥53	4 (36.4)	9 (50)	
FIGO stage			0.62
III	10 (90.9)	14 (77.8)	
IV	1 (9.1)	4 (22.2)	
Histology			0.17
Serous	7 (63.6)	15 (83.3)	
Non-serous	4 (36.4)	3 (16.7)	
Grade			1.00
1	0 (0)	0 (0)	
2	3 (27.2)	5 (27.8)	
3	4 (36.4)	9 (50)	
Unknown	4 (36.4)	4 (22.2)	
Residual tumor (cm)			0.02
≥1	7 (63.6)	3 (16.7)	
<1	4 (36.4)	15 (83.3)	
Neoadjuvant chemotherapy			1.00
No	9 (81.8)	15 (83.3)	
Yes	2 (18.2)	3 (16.7)	

-Supplementary Table 2 continued

Characteristics	Weight loss ≥10% (n=11, %)	Weight loss <10% (n=18, %)	P value
Cycles of adjuvant chemotherapy			0.36
≤6	10 (90.9)	13 (72.2)	
>6	1 (9.1)	5 (27.8)	
CA-125 (median, range, U/ml)	10.3 (1.8, 104)	8 (3.7, 27.0)	0.52
NLR (median, range)	2.15 (1.05, 41.03)	2.04 (0.56, 7.16)	0.94

FIGO, International Federation of Gynecology and Obstetrics; NLR, neutrophil to lymphocyte ratio.
